# Risk analysis of the association between different hemoglobin glycation index and poor prognosis in critical patients with coronary heart disease-A study based on the MIMIC-IV database

**DOI:** 10.1186/s12933-024-02206-1

**Published:** 2024-03-30

**Authors:** Xing Wei, Xinghua Chen, Zhipeng Zhang, Jing Wei, Ben Hu, Nv Long, Jun Feng, Chunmiao Luo

**Affiliations:** 1 Department of Cardiology, The Second People’s Hospital of Hefei, Hefei Hospital Affiliated to Anhui Medical University, Hefei, 230011 Anhui China; 2 The Fifth Clinical School of Medicine, Anhui Medical University, Hefei, 230032 Anhui China; 3 Intensive Care Unit, The Second People’s Hospital of Hefei, Hefei Hospital Affiliated to Anhui Medical University, Hefei, 230011 China

**Keywords:** Coronary heart disease, Hemoglobin glycation index, MIMIC-IV, All-cause mortality, ICU

## Abstract

**Background:**

The hemoglobin glycation index (HGI) is the difference between the observed and predicted values of glycosylated hemoglobin (HbA1c), which is closely associated with a variety of poor prognoses. However, there are still no studies on the correlation between HGI and poor prognosis in patients with critical coronary artery disease. The purpose of this study was to analyze the correlation between HGI and all-cause mortality in patients with critical coronary artery disease using the MIMIC-IV database.

**Methods:**

The HGI was calculated by constructing a linear regression equation between HbA1c and fasting plasma glucose (FPG). A Kaplan‒Meier survival analysis model was constructed based on the HGI quartiles to clarify the differences in all-cause mortality rates between groups, and the log-rank test was used to assess the differences between groups. The hazard ratio (HR) of HGI as a risk factor for outcome events was assessed using the Cox proportional risk model and restricted cubic spline (RCS), with the Q2 group serving as the reference group.

**Results:**

A total of 5260 patients were included in this study. The 30-day mortality rate of the patients was 4.94% and the mortality rate within 365 days was 13.12%. A low HGI was significantly associated with 30-day mortality (HR, 1.96; 95% CI, (1.38, 2.78); *P* < 0.001) and 365-day mortality (HR, 1.48; 95% CI, (1.19, 1.85); *P* < 0.001) in patients with critical coronary artery disease in the completely adjusted Cox proportional risk model. In addition, high levels of HGI were associated with 365-day mortality (HR, 1.31; 95% CI, (1.02, 1.69); *P* < 0.05). RCS analysis revealed a U-shaped relationship between HGI and outcome events. According to the stratified analysis, the interaction test revealed that the correlation between HGI and outcome events remained stable.

**Conclusion:**

There was a significant correlation between HGI and all-cause mortality in patients with critical coronary artery disease, particularly in those with low HGI. HGI can be used as a potential indicator for assessing the short- and long-term risk of mortality in such patients.

**Supplementary Information:**

The online version contains supplementary material available at 10.1186/s12933-024-02206-1.

## Introduction

Currently, the global burden of coronary artery disease (CAD) remains a critical global public health problem that requires attention, despite increasing improvements in preventive measures and treatment options [1]. Among critically ill patients in the intensive care unit (ICU), patients with coronary artery disease are likely to require treatment in the ICU after cardiac surgery, which is one of the main reasons for admission to the ICU; moreover, the mortality rate due to exacerbation of chronic cardiovascular disease within 1 year is 16.1%, which is the second leading cause of death after malignant tumours [2, 3]. Prognostic management of patients with critical coronary artery disease is a current medical priority that requires attention; however, few current studies have evaluated the prognosis of patients with critical coronary artery disease.

Diabetes is one of the underlying diseases of coronary heart disease, and studies have revealed that patients with diabetes have a significantly higher risk of diffuse coronary atherosclerosis and fatal coronary heart disease than patients without diabetes [4–6]. A previous cohort study showed that metabolic syndrome is a key risk factor for an increased risk of death from CAD, and that diabetes is one of the key components of metabolic syndrome [7]. Several studies have shown that stabilized and regulated blood glucose levels have long-lasting benefits for CAD patients [8, 9]. Glycosylated hemoglobin (HbA1c), utilized in the diagnosis and management of diabetes mellitus, reflects an individual’s average blood glucose over a three-month period and is currently the most commonly used surrogate marker of the effectiveness of glucose-lowering interventions [10, 11]. However, there is evidence that HbA1c is consistently higher or lower than fasting plasma glucose (FPG) levels in some populations [12]. There are several independent effects of the mean erythrocyte lifespan, differences in cell membrane glucose transmembrane gradients, enzyme abnormalities, and genetic factors on HbA1c [13–15].

The hemoglobin glycation index (HGI) quantifies changes in the relationship between HbA1c and plasma glucose concentration [16]. The HGI was defined as the difference between the observed HbA1c and the predicted HbA1c in a linear regression equation fitted according to FPG [17]. Several studies have shown that HGI predicts the risk of diabetes complications, including mortality, cardiovascular disease, and microvascular complications [17, 18]. In the above studies, a high HGI was strongly associated with major adverse cardiovascular events in the experimental population. However, several studies have shown that patients in the subgroup with low HGI have a higher risk of adverse outcomes [18, 19]. Glycaemic control in critically ill CHD patients under intensive care is particularly important for their survival [20]. Previous studies have shown that the HGI can be a relatively intuitive reflection of glycaemic variability in patients [21]. There are relatively few studies on glycaemic variability in patients with critical coronary artery disease. Exploring the correlation between HGI and the prognosis of patients with critical coronary artery disease is beneficial for understanding the significance of the relationship between glycaemic variability and long-term survival. Additionally, HGI is related not only to HbA1c, which can reflect the long-term glycaemic control of patients but also to the immediate FPG of patients. Enhancing the management of patients’ HGI also improves their short-term glycaemic control. Evaluating whether HGI is a valid prognostic risk stratifier for patients with critical CHD may help identify patients at high risk of all-cause mortality who are candidates for early surveillance and intervention. In contrast, few previous studies have evaluated the relationship between HGI and prognosis in patients with critical coronary heart disease. Therefore, in the present study, the Medical Information Mart for Intensive Care IV (MIMIC-IV) was used to construct linear regression equations to calculate the HGI and analyse the correlation between the HGI and adverse outcomes in patients with critical coronary artery disease.

## Methods and materials

### Study population

In the present study, the authors retrospectively retrieved data on patients with coronary artery disease from the MIMIC-IV database, which is a large database developed and managed by the Laboratory of Computational Physiology at the Massachusetts Institute of Technology. This database contains medical information about patients admitted to the intensive care unit at Beth Israel Deaconess Medical Center [22]. One of the authors of this study obtained permission to access this dataset and extract the relevant data. The database was approved for research use by the review committee of the Massachusetts Institute of Technology and Beth Israel Deaconess Medical Center, and a waiver of informed consent was granted.

Consistent with previous studies [23], the diagnoses of CHD, heart failure, hypertension, atrial fibrillation, hypertension, diabetes mellitus, and CKD-5 in this study were based on International Classification of Diseases ICD-9 and ICD-10 codes (the ICD-9 and ICD-10 codes for all diseases are shown in Additional Table S1). In this investigation, we enrolled 15,298 patients with critical coronary artery disease who were first admitted to the intensive care unit from 2008 to 2019. We excluded 9731 patients who lacked data on their HbA1c level and 307 patients who lacked data on their fasting glucose; ultimately, 5260 patients with critical coronary artery disease were included.

### Data extractions

PostgresSQL (version 13.7.2) and Navicate Premium (version 16) software were used to extract information through the running Structured Query Language (SQL). Potential confounding variables included in this study included the following: 1, baseline demographic information: age, gender, BMI, 2, comorbidities: hypertension, diabetes, atrial fibrillation(AF), chronic kidney disease (CKD) stage 5, acute myocardial infarction(AMI), acute heart failure(AHF), 3, history of coronary artery surgery: coronary artery bypass grafting(CABG), percutaneous transluminal coronary angioplasty(PTCA), 4, treatments: antiplatelet therapy (including doublet antiplatelet and mono-antiplatelet), lipid-regulating drugs (including statins and fibrates), angiotensin-converting enzyme inhibition (ACEI)/ angiotensin receptor blocker (ARB), β-blockers, insulin, invasive ventilation, 5, laboratory parameters: white blood cells(WBC), red blood cells(RBC), hemoglobin(HGB), platelets(PLT), blood creatinine, 6, severity of illness scores: Acute Physiology Score III (APSIII), and Sepsis-Organ Failure Assessment Score (SOFA). None of the above variables were missing except for serologic indicators. We used the random forest method for multiple interpolation for all serologic indicators, considering that the missing values did not exceed 5%.

### Definition of exposure variables and outcome events

**Fig. 1:**
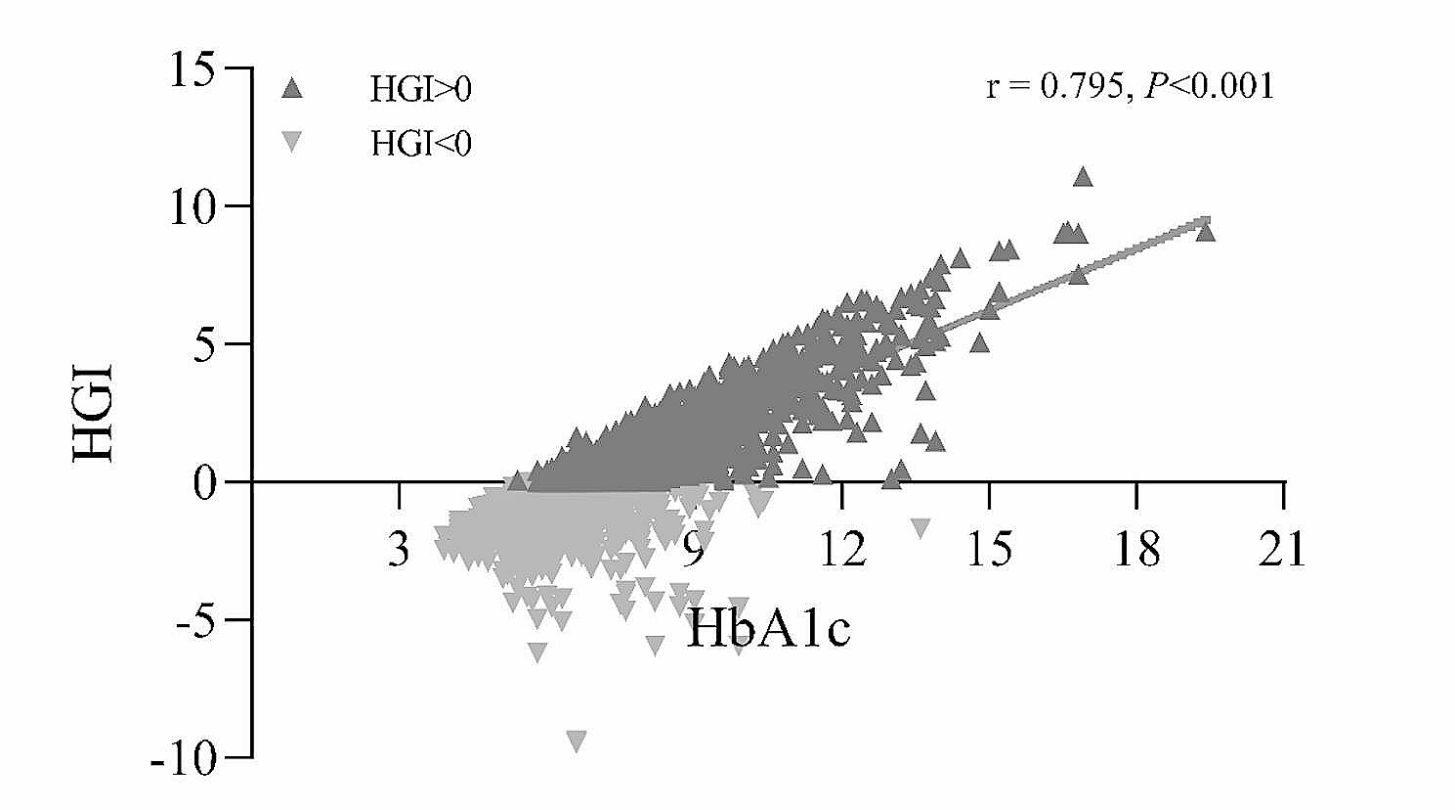
The correlation between HGI and HbA1c

A linear regression model between FPG and HbA1c was developed based on all patients included in this study. Based on this, the predicted HbA1c was calculated (predicted HbA1c = 0.013*FPG + 4.804) and subsequently the difference between the observed and predicted values of HbA1c was calculated as the HGI^16^. The correlation between HGI and HbA1c is shown in Fig. 1.

The primary outcome event in this study was all-cause mortality within 365 days after patient admission, and the secondary outcome event was defined as all-cause mortality within 30 days after admission.

### Statistical analysis

HGI quartiles were used to categorize the study population into four groups: Q1 (*n* = 1315, HGI ≤ -0.77), Q2 (*n* = 1314, -0.77 < HGI ≤ -0.37), Q3 (*n* = 1316, -0.37 < HGI ≤ 0.25), and Q4 (*n* = 1315, HGI > 0.25). Categorical variables were expressed as percentages, and comparisons between groups were made using the chi-square test; all continuous numerical variables were expressed as medians (quartiles) after a normality test, and comparisons between groups were made using the nonparametric rank-sum test. The hazard ratio (HR) of HGI as a risk factor for outcome events was assessed using the Cox proportional risk model with the Q2 group as the reference group. In the multivariate Cox regression model, age, gender, BMI, PTCA, CABG, AMI, AHF, AF, hypertension, diabetes, CKD5, invasive ventilation therapy, antiplatelet therapy, lipid-regulating drugs, ACEI/ARB, β-blockers, and insulin were included as confounders and tested for trend. Kaplan‒Meier survival analysis based on HGI quartiles was used and the log-rank test was used to assess differences between groups. Restricted cubic spline (RCS) curves were used to explore the association between HGI and outcome events, and a threshold effect model was developed to analyze the inflection point of HGI. In addition, we further performed subgroup analyses, which were used to verify the robustness of the results. Statistical analysis for this study was performed using R studio (version R4.2.3) and EmpowerStats (version 4.1). A two-sided *P* value < 0.05 was regarded as statistically significant.

## Results

### Comparison of patients’ baseline information

This research included 5260 patients with critical coronary artery disease, including 3748 male patients (71.3%, 68.8 ± 11.4 years) and 1512 female patients (28.7%, 72.8 ± 11.1 years), of whom 690 patients experienced a fatal event during the 365-day follow-up period. The baseline data based on the HGI quartiles are shown in Table 1. Compared to the low-HGI group, the high-HGI group had a lower percentage of young male patients, had a higher BMI, and had a higher CABG percentage, PLT count, and creatinine level. However, the percentages of patients with AMI, AHF, AF, CKD5, and high hemoglobin levels were larger in the low HGI group. In addition, we found that disease severity scores (SOFA score, APSIII score) and WBC counts were higher in the high HGI group and the low HGI group than in the median HGI group.

**Table 1:** Comparison of patients’ baseline information

HGI quartile	Q1 (<-0.77)	Q2(-0.77, -0.37)	Q3(-0.37, 0.25)	Q4 (> 0.25)	P-value
N	1315	1314	1316	1315	
Age (years)	70.85 (62.11–78.83)	70.49 (62.26–78.35)	72.32 (64.32–79.85)	68.17 (60.70–76.13)	< 0.001
Gender (%)					< 0.001
Male	957 (72.78%)	989 (75.27%)	906 (68.84%)	896 (68.14%)	
Female	358 (27.22%)	3\5 (24.73%)	410 (31.16%)	419 (31.86%)	
BMI (kg/m^2^)	28.16(25.03–31.59)	28.19 (25.26–31.19)	28.54 (25.26–31.96)	29.58 (26.54–33.15)	< 0.001
CABG (%)					0.025
No	1203 (91.48%)	1218 (92.69%)	1197 (90.96%)	1175 (89.35%)	
Yes	112 (8.52%)	96 (7.31%)	119 (9.04%)	140 (10.65%)	
PTCA (%)					0.060
No	1070 (81.37%)	1065 (81.05%)	1047 (79.56%)	1020 (77.57%)	
Yes	245 (18.63%)	249 (18.95%)	269 (20.44%)	295 (22.43%)	
AMI (%)					< 0.001
No	892 (67.83%)	1000 (76.10%)	1048 (79.64%)	981 (74.60%)	
Yes	423 (32.17%)	314 (23.90%)	268 (20.36%)	334 (25.40%)	
AHF (%)					< 0.001
No	1004 (76.35%)	1102 (83.87%)	1059 (80.47%)	1024 (77.87%)	
Yes	311 (23.65%)	212 (16.13%)	257 (19.53%)	291 (22.13%)	
AF (%)					< 0.001
No	776 (59.01%)	816 (62.10%)	760 (57.75%)	886 (67.38%)	
Yes	539 (40.99%)	498 (37.90%)	556 (42.25%)	429 (32.62%)	
Hypertension (%)					< 0.001
No	678 (51.56%)	565 (43.00%)	566 (43.01%)	652 (49.58%)	
Yes	637 (48.44%)	749 (57.00%)	750 (56.99%)	663 (50.42%)	
Diabetes (%)					< 0.001
No	971 (73.84%)	1026 (78.08%)	763 (57.98%)	114 (8.67%)	
Yes	344 (26.16%)	288 (21.92%)	553 (42.02%)	1201 (91.33%)	
CKD5 (%)					< 0.001
No	1242 (94.45%)	1289 (98.10%)	1287 (97.80%)	1276 (97.03%)	
Yes	73 (5.55%)	25 (1.90%)	29 (2.20%)	39 (2.97%)	
Invasive ventilation (%)					0.224
No	353 (26.84%)	314 (23.90%)	331 (25.15%)	354 (26.92%)	
Yes	962 (73.16%)	1000 (76.10%)	985 (74.85%)	961 (73.08%)	
Blood lipid regulation (%)					< 0.001
No	130 (9.89%)	78 (5.94%)	82 (6.23%)	78 (5.93%)	
Yes	1185 (90.11%)	1236 (94.06%)	1234 (93.77%)	1237 (94.07%)	
Antiplatelet (%)					0.163
No	61 (4.64%)	39 (2.97%)	44 (3.34%)	49 (3.73%)	
Single antiplatelet	914 (69.51%)	923 (70.24%)	918 (69.76%)	884 (67.22%)	
Double antiplatelet	340 (25.86%)	352 (26.79%)	354 (26.90%)	382 (29.05%)	
ACEI/ARB (%)					< 0.001
No	688 (52.32%)	634 (48.25%)	547 (41.57%)	499 (37.95%)	
Yes	627 (47.68%)	680 (51.75%)	769 (58.43%)	816 (62.05%)	
β-blockers (%)					0.003
No	146 (11.10%)	107 (8.14%)	108 (8.21%)	150 (11.41%)	
Yes	1169 (88.90%)	1207 (91.86%)	1208 (91.79%)	1165 (88.59%)	
Insulin (%)					< 0.001
No	491 (37.34%)	528 (40.18%)	445 (33.81%)	100 (7.60%)	
Yes	824 (62.66%)	786 (59.82%)	871 (66.19%)	1215 (92.40%)	
SOFA	5.00 (3.00–7.00)	4.00 (2.00–6.00)	4.00 (2.00–7.00)	5.00 (3.00–7.00)	< 0.001
APSIII	37.00 (27.50–53.00)	34.00 (26.00-45.75)	35.00 (28.00–47.00)	40.00 (31.00–51.00)	< 0.001
WBC (×10^9^/L)	9.50 (7.00-13.30)	9.10 (7.00–12.13)	9.20 (7.00–12.10)	9.50 (7.40–12.50)	0.005
RBC (×10^9^/L)	3.94 (3.24–4.46)	3.99 (3.35–4.50)	3.99 (3.37–4.48)	3.93 (3.33–4.50)	0.131
PLT (×10^9^/L)	193.00 (155.00-245.00)	202.00 (159.00-245.00)	201.00 (161.00-245.92)	206.00 (161.00-261.00)	0.001
HGB (g/L)	12.00 (9.90–13.70)	12.18 (10.20–13.80)	11.90 (10.00–13.40)	11.40 (9.80–13.30)	< 0.001
blood creatinine (mg/dL)	1.00 (0.80–1.40)	1.00 (0.80–1.20)	1.00 (0.80–1.30)	1.10 (0.90–1.40)	< 0.001
FPG (mg/dL)	131.00 (109.00–168.00)	109.00 (98.00–127.80)	109.00 (97.00–134.00)	142.00 (106.00–182.00)	< 0.001

Continuous numerical variables are expressed as medians (interquartile spacing) and categorical variables are expressed as numbers (percentages)

CABG: coronary artery bypass grafting, PTCA: Percutaneous transluminal coronary angioplasty, AMI: acute myocardial infarction, AHF: acute heart failure, AF: atrial fibrillation, CKD5: chronic kidney disease stage 5, ACEI: angiotensin-converting enzyme inhibition, ARB: angiotensin receptor blocker, SOFA: Sepsis-Organ Failure Assessment Score, APSIII: Acute Physiology Score III, WBC: white blood cells, RBC: red blood cells, PLT: platelets, HGB: hemoglobin, FPG: fasting plasma glucose

### Survival analysis

**Fig. 2:**
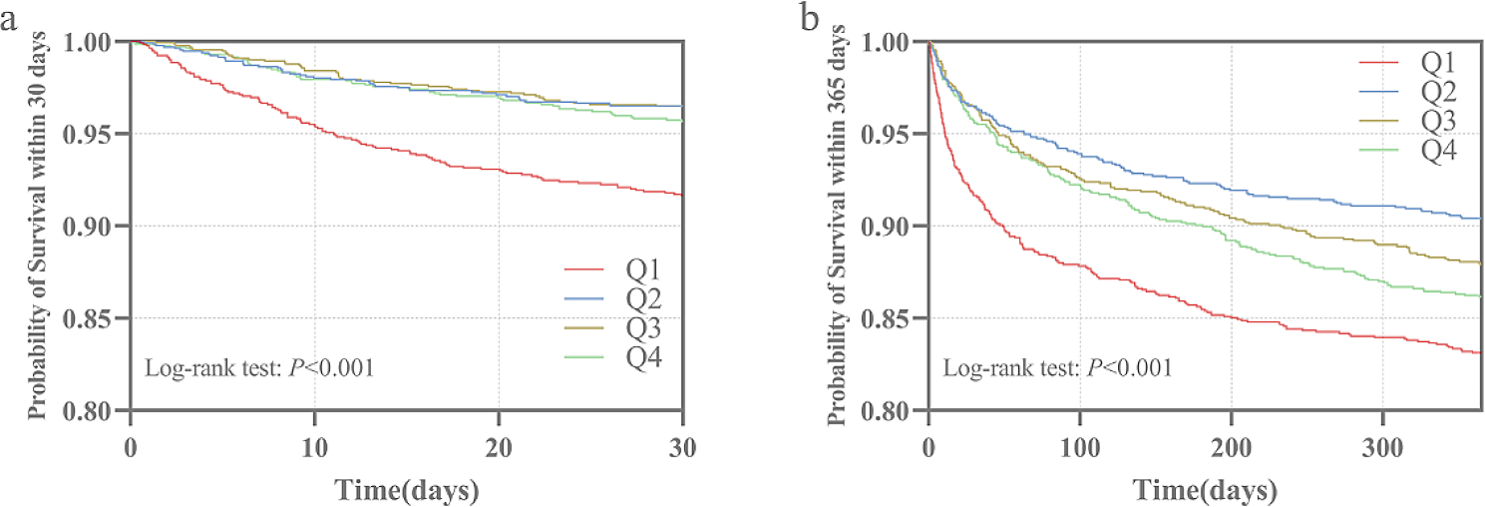
Kaplan-Meier all-cause mortality survival analysis curve **a**: showing comparison of mortality within 30 days between groups, **b**: showing comparison of mortality within 365 days between groups

We compared the incidence of the primary outcome between groups using Kaplan‒Meier survival analysis curves based on the HGI quartiles (as shown in Fig. 2). The rate of mortality within 30 days was significantly higher in the Q1 group than in the other groups (log-rank *P* < 0.001) (Fig. 2a). The one-year mortality rate was significantly higher in Groups Q1 and Q4 than that in Groups Q2 and Q3, and the difference between the groups was significant (log-rank *P* < 0.001), which indicated that both high and low HGI were detrimental to the long-term survival of patients with coronary artery disease and that low HGI was particularly predominant (Fig. 2b).

### Correlation of the HGI with outcome events

In the comparison of patients’ baseline information, we found that the Q2 group (-0.77 < HGI≤-0.37) had the lowest mortality rate compared to the remaining groups. Based on the above, we analyzed the correlation between HGI and the primary outcome by developing Cox proportional risk models with the Q2 group as the reference group. The results indicated that the Q1 group (HGI < -0.77) was associated with both the primary outcome event (Q1 vs. Q2: HR, 1.86 [1.49, 2.31] *P* < 0.001) and the secondary outcome (Q1 vs. Q2: HR, 2.46 [1.74, 3.46] *P* < 0.001) in the Cox proportional risk model unadjusted for confounders. We observed that HGI remained associated with 365-day mortality (Q1 vs. Q2: HR, 1.48 [1.19, 1.85] *P* < 0. 001), and 30-day mortality (Q1 vs. Q2: HR, 1.96 [1.38, 2.78] *P* < 0.001) in models completely adjusted for confounders. In addition, we identified a correlation between the Q4 group (HGI > 0.25) and 365-day mortality (Q1 vs. Q2: HR, 1.31 [1.02, 1.69] *P* < 0.05), but not between the Q4 group (HGI > 0.25) and 30-day mortality, in the model completely adjusted for confounders (Table 2).

**Table:** **Table 2**

HGI group	Non-adjusted	Adjust I	Adjust II
All-cause mortality within 30 days			
HGI (<-0.77)	2.46 (1.74, 3.46) ***	2.43 (1.73, 3.44) ***	1.96 (1.38, 2.78) ***
HGI (-0.77, -0.37)	ref	ref	ref
HGI (-0.37, 0.25)	1.02 (0.68, 1.53)	0.93 (0.62, 1.40)	1.06 (0.70, 1.59)
HGI (> 0.25)	1.24 (0.84, 1.83)	1.37 (0.93, 2.03)	1.31 (0.86, 2.00)
HGI group trend	< 0.001	0.004	0.038
All-cause mortality within 365 days			
HGI (<-0.77)	1.86(1.49, 2.31) ***	1.84(1.48, 2.29) ***	1.48(1.19, 1.85) ***
HGI (-0.77, -0.37)	ref	ref	ref
HGI (-0.37, 0.25)	1.28 (1.01, 1.61) *	1.15 (0.91, 1.46)	1.13 (0.89, 1.43)
HGI (> 0.25)	1.47 (1.17, 1.84) ***	1.59 (1.27, 2.00) ***	1.31 (1.02, 1.69) *
HGI group trend	0.367	0.947	0.548

Adjust I: Adjusted for age, gender, BMI.Adjust II: Adjusted for age, gender, BMI, CABG, PTCA, AMI, AHF, AF, Hypertension, Diabetes, Invasive ventilation, Blood lipid regulation, Antiplatelet, ACEI/ARB, β-blockers, Insulin

*P*-value *P < 0.05 **P < 0.01 ***P < 0.001

**Fig. 3:**
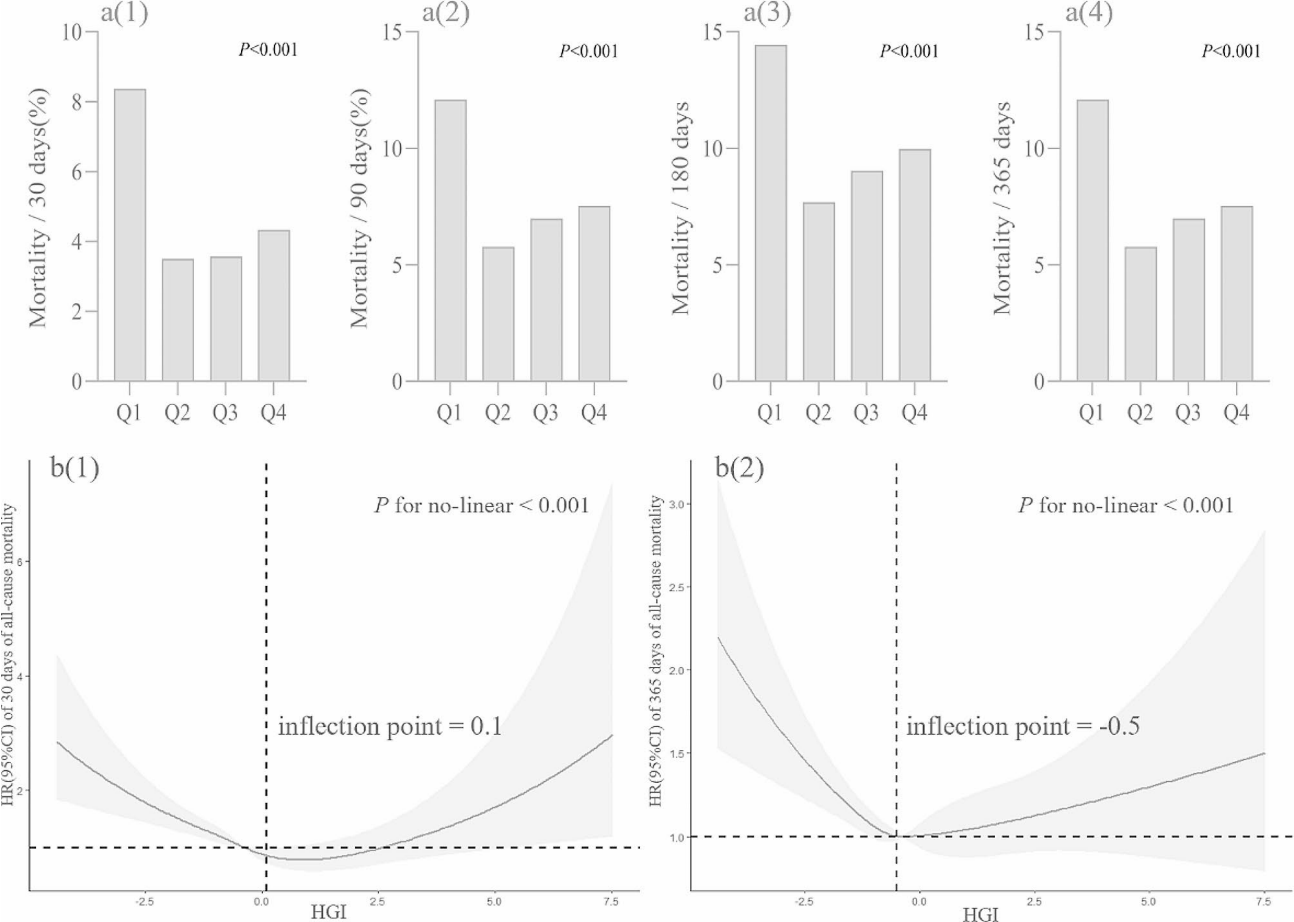
Correlation between HGI and all-cause mortality hazard ratio in patients with critical coronary heart disease. **a**: Comparison of all-cause mortality between groups based on HGI quartiles. **b**: Restricted cubic spline curve for HGI hazard ratio. Vertical dashed lines indicate inflection points, dark gray lines indicate fully adjusted risk ratios, shaded areas indicate 95% confidence intervals, and horizontal dashed hazard ratio 1. **b**(**1**): Restricted Cubic Spline Curve for the mortality rate of patients within 30 days, **b**(**2**): Restricted Cubic Spline Curve for the mortality rate of patients within 365 days

As shown in Fig. 3.a, the Q1 group had the highest mortality rate, followed by the Q4 group. Therefore, we concluded that both high level HGI and low level HGI are risk factors for patients with critical coronary heart disease. Subsequently, we modelled the RCS. The results showed a “U” relationship between HGI and the risk ratio of death in patients with critical coronary artery disease (Fig. 3b (1) and Fig. 3b (2)). In the threshold effect analysis, we found HGI inflection points of 0.1 and − 0.5 for the primary and secondary outcomes, respectively (Table 3). In addition, we further analyzed the relationship between HGI and all-cause mortality in CHD patients in both diabetic and nondiabetic CHD patients using RCS. The results showed a nonlinear relationship (U-shaped curve) between HGI and all-cause mortality in CHD patients, both for diabetic and nondiabetic patients (Additional Figure S1).

**Table 3:** Threshold effect analysis

	Mortality within 30 days	Mortality within 365 days
Standard Linear Regression Model	0.9 (0.8, 1.0) 0.047	1.0 (0.9, 1.0) 0.237
Two-stage regression models		
inflection point (K)	0.1	-0.5
<K	0.8 (0.7, 0.8) < 0.001	0.8 (0.7, 0.9) < 0.001
>K	1.2 (1.0, 1.3) 0.021	1.1 (1.0, 1.1) 0.099
log-likelihood ratio test	< 0.001	< 0.001

### Subgroup analysis

**Fig. 4:**
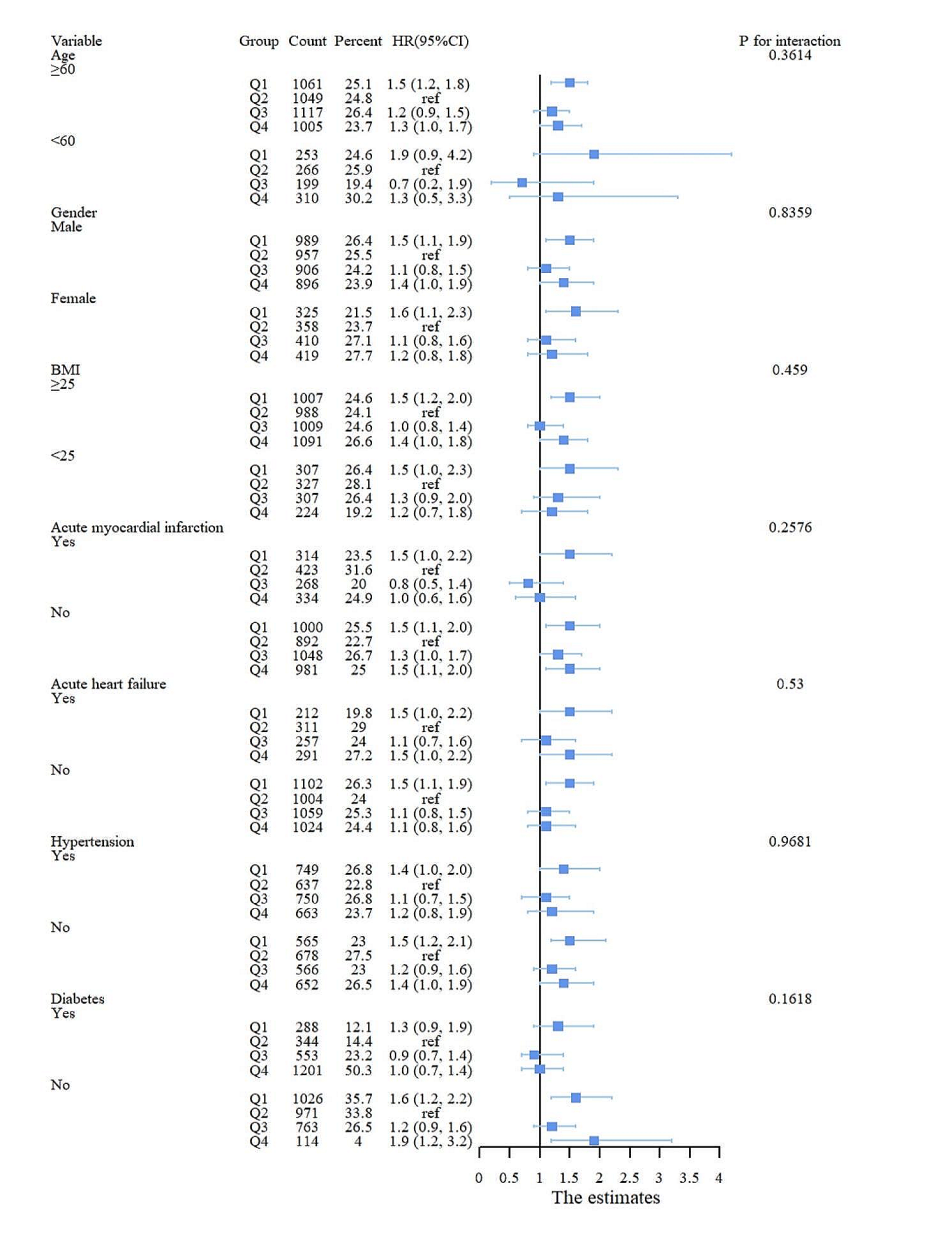
Subgroup analysis with 365-day mortality as the outcome event

**Fig. 5:**
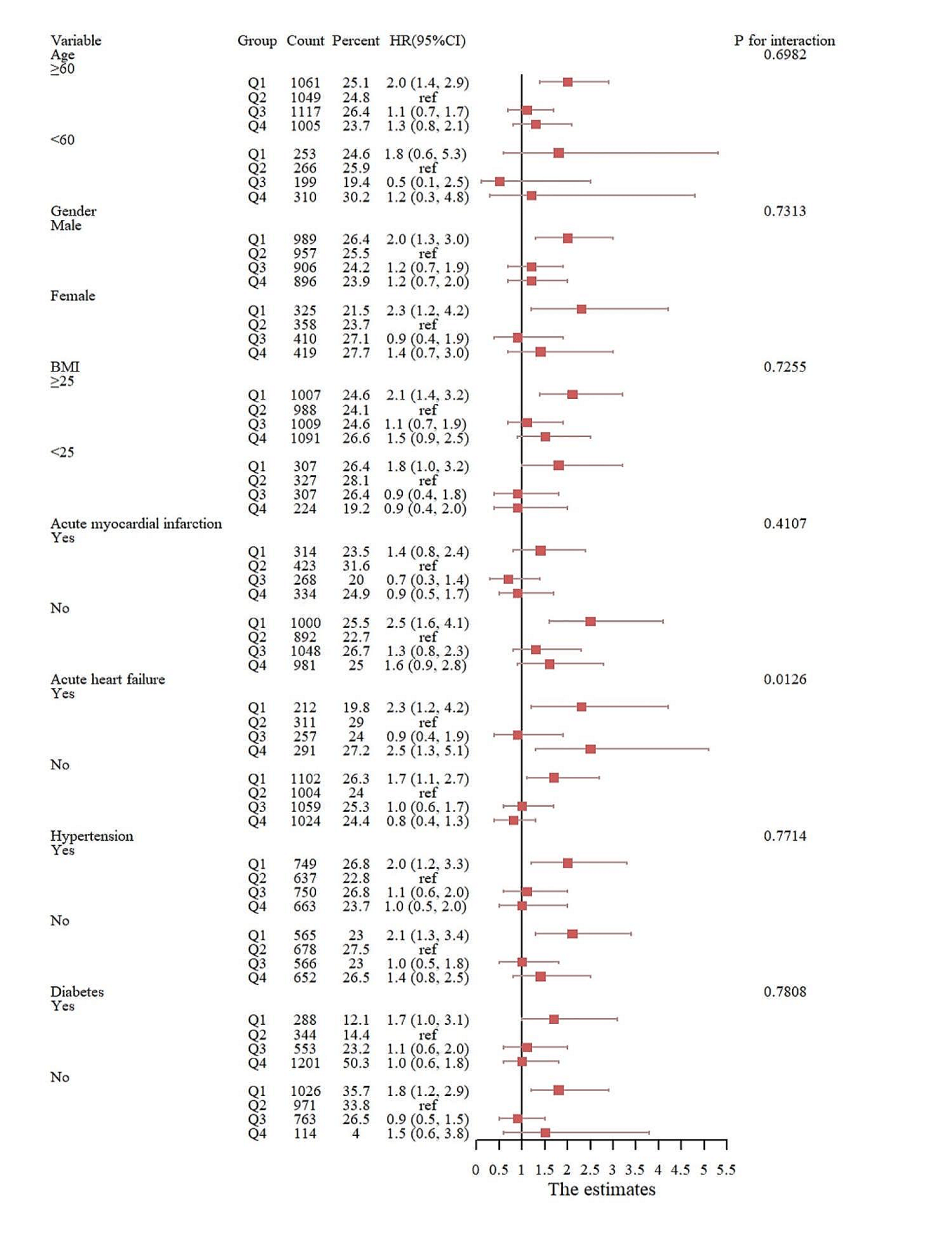
Subgroup analysis with 30-day mortality as the outcome event

In addition, we performed risk subgroup analyses of patient outcome events according to age, gender, BMI, AMI, AHF, hypertension, and diabetes. We observed that in a subgroup analysis with 365-day mortality as the outcome event, low levels of HGI (<-0.77) in the remaining subgroups were closely associated with primary 365-day mortality in patients with critical coronary artery disease, with the exception of patients aged < 60 years, those with AMI, those with diabetes, and those with hypertension. High levels of HGI (> 0.25) were significantly associated with age > 60 years (HR, 1.3 (1.0, 1.7) *P* = 0.043), BMI > 25 (HR, 1.4 (1.0, 1.8) *P* = 0.044), non-AMI (HR, 1.5 (1.1, 2.0) *P* = 0.013), and non-diabetes (HR, 1.9 (1.2, 3.2) *P* = 0.009) (Fig. 4). According to the subgroup analysis with 30-day mortality as the outcome event, low HGI (<-0.77) in the remaining subgroups was strongly associated with primary 30-day mortality in patients with critical coronary artery disease, with the exception of patients < 60 years of age, those with a BMI < 25, and those with diabetes. High HGI (> 0.25) was associated with short-term mortality only in the acute myocardial infarction subgroup (Fig. 5).

## Discussion

Several previous investigations have shown that HGI is strongly associated with adverse cardiovascular events [18], kidney injury [24, 25], and nonalcoholic fatty liver disease [26]. However, both high HGI and low HGI appear to be associated with poor prognosis to varying degrees. In the present study, we aimed to assess the correlation between HGI and the prognosis of patients with critical coronary artery disease by utilizing the MIMIC-IV database. The results suggest that both low and high HGI correlate with poor prognosis in patients with critical coronary artery disease in terms of long-term patient outcomes. However, we did not observe any consistency results in patients’ short-term outcomes, as low HGI was significantly associated with short-term outcomes, and high HGI was not significantly associated with short-term outcomes. We found a “U” relationship between HGI and the hazard ratio of outcome events in the RCS-based analyses, and the inflection points were all near zero, which is consistent with the results of the analyses described above. Additionally, considering that this U-shaped relationship may be influenced by the percentage of patients with diabetes, we performed an RCS analysis of the diabetes subgroup. Subsequently, we found that this U-shaped relationship remained robust for both diabetic and nondiabetic patients. Therefore, HGI may be an independent risk factor for patients with critical coronary artery disease. In clinical work, constructing linear regression models to calculate the HGI with a large sample of patients is necessary.

HbA1c is formed by the nonenzymatic reaction of intracellular HbA1 with glucose, and there is some discrepancy between actual and predicted levels, but the mechanism has not yet been clarified [27]. There was significant interindividual variation in the association between HbA1c and FPG, which could be caused by any factor capable of influencing the process of glucose metabolism, and HGI quantified this variation in HbA1c. The HGI appears to reflect interindividual glycemic variability within different populations as an important indicator of the risk of identifying microvascular complications, which may contribute to the development of complications. In the Diabetes Control and Complications Trial (DCCT), researchers divided patients with type 1 diabetes into three groups, those with high, medium, and low HGI, and found that the risk of microangiopathy of the retina and kidney vasculature increased 3- and 6-fold, respectively, with increasing HGI [28]. Therefore, identifying other factors or mechanisms that contribute to the biological variability of HbA1c without taking into account the effect of fluctuations in FPG may lead to the development of new therapies to control the development of diabetic microangiopathy. However, the impact of this variability on cardiovascular disease and mortality is still somewhat controversial [29]. To further investigate the correlation between HGI and cardiovascular disease and its prognosis, this study clarified the U-shaped association between HGI and poor prognosis in patients with critical CAD using the MIMIC-IV database.

The onset and progression of CAD are closely linked to fluctuations in blood glucose levels [30]. Tight glycemic regulation (< 7.8 mmol/L) and large glycemic fluctuations were independently associated with adverse in-hospital outcomes in diabetic patients undergoing coronary artery bypass grafting and extracorporeal circulation in a large cohort study [31]. This seems to suggest that a state of too tightly controlled “low blood glucose” levels in patients with coronary artery disease leads to worse outcomes. In the Action for Cardiovascular Risk Control in Diabetes (ACCORD) trial, researchers enrolled more than 10,000 patients with type 2 diabetes and reported that intensive glycemic control treatments had greater benefits for patients with low to moderate HGI than for those with high HGI [16, 29]. In addition, intensive treatment did not reduce the incidence of CVD events but instead increased the overall mortality rate by 41% in the high HGI group [16]. In clinical practice, different glycemic control treatments should be used for patients with different HGI. However, all of the above studies were clinical studies of HGI, and it is not known why intensive glycemic therapy was not observed to benefit patients in the high-level HGI group. In this study, lower HGI were predictive of all-cause mortality in patients with critical coronary artery disease, which seems to indicate that long-term intensive glycemic therapy can provide long-lasting benefits for critically ill CHD patients with low HGI.

Under the influence of medication, FPG can change to varying degrees. After insulin treatment, FPG levels are much lower than normal levels [32]. Thus, patients treated with insulin may have a higher HGI, regardless of their level of intrinsic organismal glycosylation. A study of the association between HGI and cardiovascular disease in people with impaired glucose metabolism included a study population that had not received insulin therapy, and after adjusting for traditional CHD risk factors, they found that higher HGI were significantly associated with vascular complications [33]. In the present study, after adjusting for traditional risk factors for all-cause mortality in patients with CHD, we adjusted for insulin as well and found that both lower and higher HGI were significantly associated with all-cause mortality. Notably, the FPG levels in both the Q1 and Q4 groups in this study were significantly higher than those in the Q2 and Q3 groups. Since a high FPG is a predictor of cardiovascular events [34], it follows that relatively high FPG mediated an increased risk of all-cause mortality in patients with critical coronary artery disease in both the low and high HGI groups of this study.

Short-term stress in critically ill patients leads to a relatively acute increase in blood glucose [35]. Stress hyperglycemia can exacerbate the severity of coronary artery disease in patients with coronary artery disease in a number of ways, such as causing endothelial dysfunction and exacerbating microvascular obstruction to damage the vascular endothelium [36], which is independently associated with short-term adverse clinical outcomes [37]. Previous studies have demonstrated that stress hyperglycemia increases the inflammatory load and risk of ischaemia–reperfusion injury, both of which are strongly associated with adverse cardiovascular outcomes. In the present study, there was a statistically significant difference in leukocyte counts between the groups, which seems to indicate that either high HGI or low HGI are associated with a heavier inflammatory load. According to our subgroup analyses, a lower HGI was strongly associated with 30-day mortality in patients with critical coronary artery disease, whereas a high HGI was not significantly associated with short-term mortality. This may be related to increased blood glucose levels during stress in patients with critical coronary artery disease. Interestingly, in the diabetic subgroup, HGI did not seem to be associated with all-cause mortality in patients with critical coronary artery disease, which is different from the findings of previous studies.

In conclusion, this study revealed that HGI is significantly associated with all-cause mortality in patients with critical coronary artery disease, and increased attention should be given to the extent of glycemic changes in patients during their stay in the ICU. In addition, considering the presence of more confounding factors in patients in the IUC, it is necessary to conduct a large-scale prospective study to clarify the correlation between HGI and adverse outcomes in patients with critical coronary artery disease.

### Limitations

In this study, we extracted relevant clinical information about patients with coronary artery disease from the MIMIC-IV database; however, we may not have extracted the full clinical diagnostic information of the patients, and there may be several confounding factors affecting the incidence of all-cause mortality. Second, the association of HGI with adverse outcomes other than all-cause mortality was not considered in this study. In addition, the patient’s lipid level was found to be an independent risk factor for a poor prognosis; however, we did not include their levels in this study because of the excessive amount of missing lipid data. Finally, in our study, the HGI was calculated based on the study population and cannot be generalized to other populations, and we believe that regression models should be built on data retrieved from various large databases to calculate the HGI for each type of population.

## Conclusion

By using patient data retrieved from the MIMIC-IV database, the authors were able to reveal that HGI was nonlinearly associated with all-cause mortality in patients with critical coronary artery disease. In terms of research on HGI and CHD, the present study differed somewhat from previous studies. Previous studies have shown that a high HGI is strongly associated with adverse cardiovascular events. In the present study, a low HGI was more hazardous for CHD patients than a high HGI and was strongly associated with both short-term and long-term mortality. We considered HGI to be a good indicator of poor prognosis in patients with critical coronary artery disease and that it could be used as a potential indicator for stratifying the risk of short- and long-term mortality in such patients. In addition, considering the hyperglycemic state of critically ill patients under stress, patients with low HGI should receive extra attention during the initial ICU admission.

### Electronic supplementary material

Below is the link to the electronic supplementary material.

Supplementary Material 1

Supplementary Material 2

## Data Availability

Data is provided within the manuscript or supplementary information files.

## Abbreviations

HGI: hemoglobin glycation index
HbA1c: glycosylated hemoglobin
FPG: fasting plasma glucose
ICU: intensive care unit
MIMIC-IV: Medical Information Mart for Intensive Care IV
CAD: coronary artery disease
AMI: acute myocardial infarction
AHF: acute heart failure
AF: atrial fibrillation
CKD5: chronic kidney disease stage 5
ACEI: angiotensin-converting enzyme inhibition
ARB: angiotensin receptor blocker
WBC: white blood cells
RBC: red blood cells
HGB: hemoglobin
PLT: platelets
APSIII: Acute Physiology Score III
SOFA: Sepsis-Organ Failure Assessment Score
RCS: restricted cubic spline
HR: hazard ratio
