# Supplementary material for: Risk analysis of the association between different hemoglobin glycation index and poor prognosis in critical patients with coronary heart disease-A study based on the MIMIC-IV database
Source: Cardiovasc Diabetol. 2024 Mar 30;23:113. doi: 10.1186/s12933-024-02206-1 (PMC10981833; doi:10.1186/s12933-024-02206-1)
Supplement: Supplementary file 1 — Supplementary Material 1 [file 12933_2024_2206_MOESM1_ESM.docx]

**Additional Table S1:** **The meaning of ICD-9 and ICD-10 codes for disease**

| **Variables** | **Diagnostic code (icdCode)** | **Meaning of diagnostic code** |
| --- | --- | --- |
| **Coronary heart disease** | I2101 | ST elevation (STEMI) myocardial infarction involving left main coronary artery |
|  | I2102 | ST elevation (STEMI) myocardial infarction involving left anterior descending coronary artery |
|  | I2109 | ST elevation (STEMI) myocardial infarction involving other coronary artery of anterior wall |
|  | I2111 | ST elevation (STEMI) myocardial infarction involving right coronary artery |
|  | I2119 | ST elevation (STEMI) myocardial infarction involving other coronary artery of inferior wall |
|  | I2121 | ST elevation (STEMI) myocardial infarction involving left circumflex coronary artery |
|  | I240 | Acute coronary thrombosis not resulting in myocardial infarction |
|  | I2510 | Atherosclerotic heart disease of native coronary artery without angina pectoris |
|  | I25110 | Atherosclerotic heart disease of native coronary artery with unstable angina pectoris |
|  | I25111 | Atherosclerotic heart disease of native coronary artery with angina pectoris with documented spasm |
|  | I25118 | Atherosclerotic heart disease of native coronary artery with other forms of angina pectoris |
|  | I25119 | Atherosclerotic heart disease of native coronary artery with unspecified angina pectoris |
|  | I25700 | Atherosclerosis of coronary artery bypass graft(s), unspecified, with unstable angina pectoris |
|  | I25708 | Atherosclerosis of coronary artery bypass graft(s), unspecified, with other forms of angina pectoris |
|  | I25709 | Atherosclerosis of coronary artery bypass graft(s), unspecified, with unspecified angina pectoris |
|  | I25710 | Atherosclerosis of autologous vein coronary artery bypass graft(s) with unstable angina pectoris |
|  | I25718 | Atherosclerosis of autologous vein coronary artery bypass graft(s) with other forms of angina pectoris |
|  | I25719 | Atherosclerosis of autologous vein coronary artery bypass graft(s) with unspecified angina pectoris |
|  | I25720 | Atherosclerosis of autologous artery coronary artery bypass graft(s) with unstable angina pectoris |
|  | I25728 | Atherosclerosis of autologous artery coronary artery bypass graft(s) with other forms of angina pectoris |
|  | I25729 | Atherosclerosis of autologous artery coronary artery bypass graft(s) with unspecified angina pectoris |
|  | I25758 | Atherosclerosis of native coronary artery of transplanted heart with other forms of angina pectoris |
|  | I25790 | Atherosclerosis of other coronary artery bypass graft(s) with unstable angina pectoris |
|  | I25810 | Atherosclerosis of coronary artery bypass graft(s) without angina pectoris |
|  | I25811 | Atherosclerosis of native coronary artery of transplanted heart without angina pectoris |
|  | I2582 | Chronic total occlusion of coronary artery |
|  | I2583 | Coronary atherosclerosis due to lipid rich plaque |
|  | I2584 | Coronary atherosclerosis due to calcified coronary lesion |
|  | 41401 | Coronary atherosclerosis of native coronary artery |
|  | 41402 | Coronary atherosclerosis of autologous vein bypass graft |
|  | 41404 | Coronary atherosclerosis of artery bypass graft |
|  | 41405 | Coronary atherosclerosis of unspecified bypass graft |
|  | 41407 | Coronary atherosclerosis of bypass graft (artery) (vein) of transplanted heart |
|  | 41411 | Aneurysm of coronary vessels |
|  | 41412 | Dissection of coronary artery |
|  | 4142 | Chronic total occlusion of coronary artery |
|  | 4143 | Coronary atherosclerosis due to lipid rich plaque |
|  | 4144 | Coronary atherosclerosis due to calcified coronary lesion |
|  | 74685 | Coronary artery anomaly |
|  | 99603 | Mechanical complication due to coronary bypass graft |
|  | T82213A | Leakage of coronary artery bypass graft, initial encounter |
|  | T82218A | Other mechanical complication of coronary artery bypass graft, initial encounter |
|  | T82855A | Stenosis of coronary artery stent, initial encounter |
|  | T82855D | Stenosis of coronary artery stent, subsequent encounter |
|  | T82855S | Stenosis of coronary artery stent, sequela |
|  | V4582 | Percutaneous transluminal coronary angioplasty status |
|  | Z951 | Presence of aortocoronary bypass graft |
|  | Z955 | Presence of coronary angioplasty implant and graft |
|  | Z9861 | Coronary angioplasty status |
| **Acute myocardial infarction** | 41001 | Acute myocardial infarction of anterolateral wall, initial episode of care |
|  | 41002 | Acute myocardial infarction of anterolateral wall, subsequent episode of care |
|  | 41011 | Acute myocardial infarction of other anterior wall, initial episode of care |
|  | 41012 | Acute myocardial infarction of other anterior wall, subsequent episode of care |
|  | 41021 | Acute myocardial infarction of inferolateral wall, initial episode of care |
|  | 41022 | Acute myocardial infarction of inferolateral wall, subsequent episode of care |
|  | 41031 | Acute myocardial infarction of inferoposterior wall, initial episode of care |
|  | 41041 | Acute myocardial infarction of other inferior wall, initial episode of care |
|  | 41042 | Acute myocardial infarction of other inferior wall, subsequent episode of care |
|  | 41051 | Acute myocardial infarction of other lateral wall, initial episode of care |
|  | 41081 | Acute myocardial infarction of other specified sites, initial episode of care |
|  | 41082 | Acute myocardial infarction of other specified sites, subsequent episode of care |
|  | 41091 | Acute myocardial infarction of unspecified site, initial episode of care |
|  | 41092 | Acute myocardial infarction of unspecified site, subsequent episode of care |
|  | I2101 | ST elevation (STEMI) myocardial infarction involving left main coronary artery |
|  | I2102 | ST elevation (STEMI) myocardial infarction involving left anterior descending coronary artery |
|  | I2109 | ST elevation (STEMI) myocardial infarction involving other coronary artery of anterior wall |
|  | I2111 | ST elevation (STEMI) myocardial infarction involving right coronary artery |
|  | I2119 | ST elevation (STEMI) myocardial infarction involving other coronary artery of inferior wall |
|  | I2121 | ST elevation (STEMI) myocardial infarction involving left circumflex coronary artery |
|  | I2129 | ST elevation (STEMI) myocardial infarction involving other sites |
|  | I213 | ST elevation (STEMI) myocardial infarction of unspecified site |
|  | I214 | Non-ST elevation (NSTEMI) myocardial infarction |
|  | I219 | Acute myocardial infarction, unspecified |
|  | I21A1 | Myocardial infarction type 2 |
|  | I21A9 | Other myocardial infarction type |
|  | I222 | Subsequent non-ST elevation (NSTEMI) myocardial infarction |
| **Acute heart failure** | 42821 | Acute systolic heart failure |
|  | 42823 | Acute on chronic systolic heart failure |
|  | 42831 | Acute diastolic heart failure |
|  | 42833 | Acute on chronic diastolic heart failure |
|  | 42841 | Acute combined systolic and diastolic heart failure |
|  | 42843 | Acute on chronic combined systolic and diastolic heart failure |
|  | I5021 | Acute systolic (congestive) heart failure |
|  | I5023 | Acute on chronic systolic (congestive) heart failure |
|  | I5031 | Acute diastolic (congestive) heart failure |
|  | I5033 | Acute on chronic diastolic (congestive) heart failure |
|  | I5043 | Acute on chronic combined systolic (congestive) and diastolic (congestive) heart failure |
| **Hypertension** | 4019 | Unspecified essential hypertension |
|  | I10 | Essential (primary) hypertension |
|  | 40390 | Hypertensive chronic kidney disease, unspecified, with chronic kidney disease stage I through stage IV, or unspecified |
|  | I129 | Hypertensive chronic kidney disease with stage 1 through stage 4 chronic kidney disease, or unspecified chronic kidney disease |
|  | I110 | Hypertensive heart disease with heart failure |
|  | I130 | Hypertensive heart and chronic kidney disease with heart failure and stage 1 through stage 4 chronic kidney disease, or unspecified chronic kidney disease |
|  | 4011 | Benign essential hypertension |
|  | 40310 | Hypertensive chronic kidney disease, benign, with chronic kidney disease stage I through stage IV, or unspecified |
|  | I161 | Hypertensive emergency |
|  | 4010 | Malignant essential hypertension |
|  | 40491 | Hypertensive heart and chronic kidney disease, unspecified, with heart failure and with chronic kidney disease stage I through stage IV, or unspecified |
|  | 40291 | Unspecified hypertensive heart disease with heart failure |
|  | I119 | Hypertensive heart disease without heart failure |
|  | 40290 | Unspecified hypertensive heart disease without heart failure |
| **Diabetes** | 25000 | Diabetes mellitus without mention of complication, type II or unspecified type, not stated as uncontrolled |
|  | E119 | Type 2 diabetes mellitus without complications |
|  | E1165 | Type 2 diabetes mellitus with hyperglycemia |
|  | E1122 | Type 2 diabetes mellitus with diabetic chronic kidney disease |
|  | 3572 | Polyneuropathy in diabetes |
|  | 25060 | Diabetes with neurological manifestations, type II or unspecified type, not stated as uncontrolled |
|  | E1165 | Type 2 diabetes mellitus with hyperglycemia |
|  | 36201 | Background diabetic retinopathy |
|  | 25040 | Diabetes with renal manifestations, type II or unspecified type, not stated as uncontrolled |
|  | E1140 | Type 2 diabetes mellitus with diabetic neuropathy, unspecified |
|  | 25050 | Diabetes with ophthalmic manifestations, type II or unspecified type, not stated as uncontrolled |
|  | E1151 | Type 2 diabetes mellitus with diabetic peripheral angiopathy without gangrene |
|  | 25002 | Diabetes mellitus without mention of complication, type II or unspecified type, uncontrolled |
|  | 25080 | Diabetes with other specified manifestations, type II or unspecified type, not stated as uncontrolled |
|  | E11319 | Type 2 diabetes mellitus with unspecified diabetic retinopathy without macular edema |
|  | E1142 | Type 2 diabetes mellitus with diabetic polyneuropathy |
|  | E1121 | Type 2 diabetes mellitus with diabetic nephropathy |
|  | 25061 | Diabetes with neurological manifestations, type I [juvenile type], not stated as uncontrolled |
|  | 25062 | Diabetes with neurological manifestations, type II or unspecified type, uncontrolled |
|  | 25001 | Diabetes mellitus without mention of complication, type I [juvenile type], not stated as uncontrolled |
|  | 25051 | Diabetes with ophthalmic manifestations, type I [juvenile type], not stated as uncontrolled |
|  | 25041 | Diabetes with renal manifestations, type I [juvenile type], not stated as uncontrolled |
|  | 25063 | Diabetes with neurological manifestations, type I [juvenile type], uncontrolled |
|  | E10319 | Type 1 diabetes mellitus with unspecified diabetic retinopathy without macular edema |
|  | E1065 | Type 1 diabetes mellitus with hyperglycemia |
|  | E1169 | Type 2 diabetes mellitus with other specified complication |
|  | 25013 | Diabetes with ketoacidosis, type I [juvenile type], uncontrolled |
| **CKD-5** | 5856 | End stage renal disease |
|  | N186 | End stage renal disease |
|  | 40311 | Hypertensive chronic kidney disease, benign, with chronic kidney disease stage V or end stage renal disease |
|  | 40391 | Hypertensive chronic kidney disease, unspecified, with chronic kidney disease stage V or end stage renal disease |
|  | 40413 | Hypertensive heart and chronic kidney disease, benign, with heart failure and chronic kidney disease stage V or end stage renal disease |
|  | 40493 | Hypertensive heart and chronic kidney disease, unspecified, with heart failure and chronic kidney disease stage V or end stage renal disease |
|  | I120 | Hypertensive chronic kidney disease with stage 5 chronic kidney disease or end stage renal disease |
|  | I1311 | Hypertensive heart and chronic kidney disease without heart failure, with stage 5 chronic kidney disease, or end stage renal disease |
|  | I132 | Hypertensive heart and chronic kidney disease with heart failure and with stage 5 chronic kidney disease, or end stage renal disease |

**Additional Figure S1: RCS analysis of diabetes subgroups**


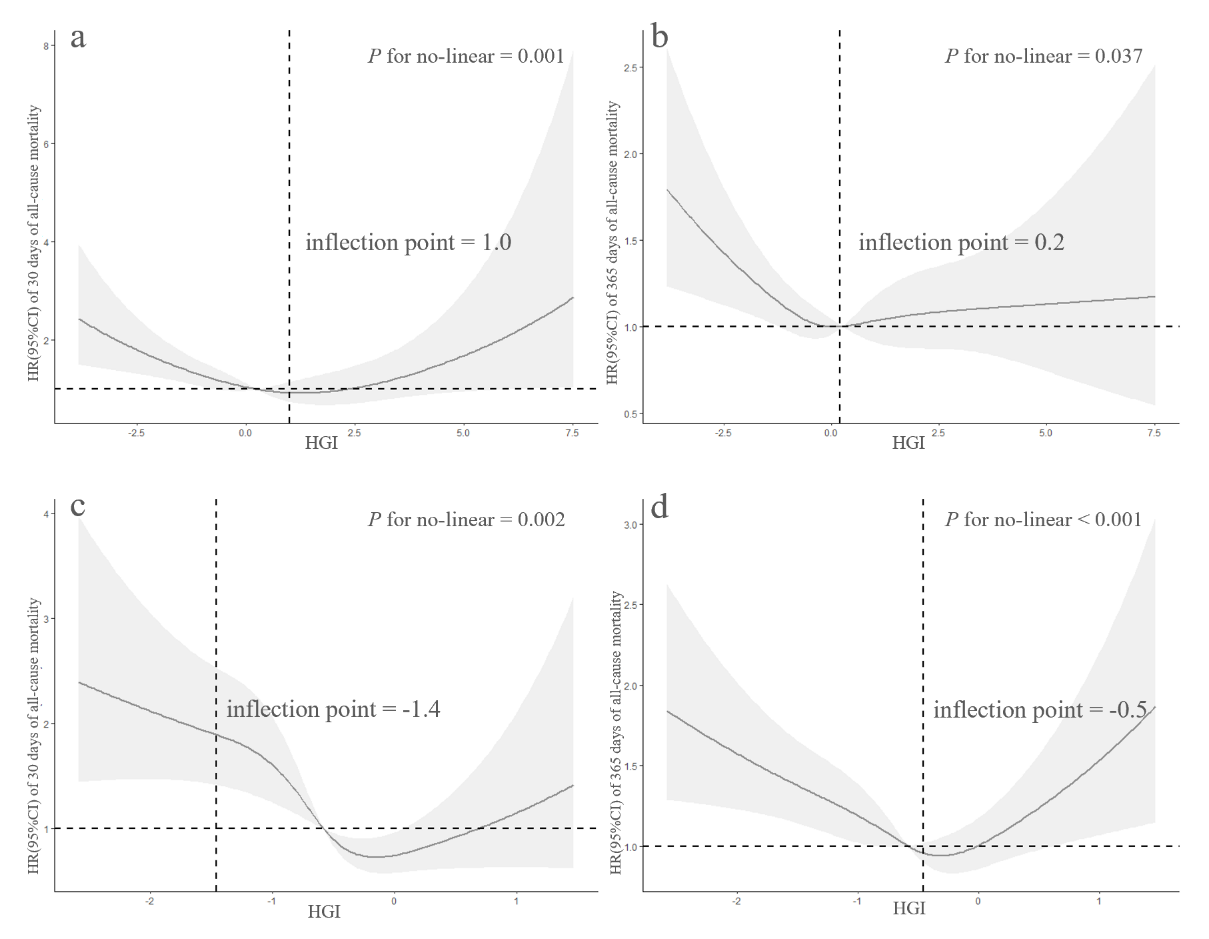


**a**: Restricted cubic spline curve for 30-day mortality in diabetic patients, **b**: Restricted cubic spline curve for 365-day mortality in diabetic patients, **c**: Restricted cubic spline curve for 30-day mortality in nondiabetic patients, **d**: Restricted cubic spline curve for 365-day mortality in nondiabetic patients.
